# From multi-level stressors to quiet quitting: the mediating role of job burnout among vocational college lecturers

**DOI:** 10.3389/fpsyg.2026.1815670

**Published:** 2026-05-07

**Authors:** Jing Zhou, Hooi Sin Soo, Azelin Binti Aziz

**Affiliations:** 1 Guizhou Communications Polytechnic University, Guiyang, Guizhou Province, China; 2 School of Business Management, Universiti Utara Malaysia, Sintok, Kedah, Malaysia

**Keywords:** conservation of resources theory, job burnout, person–organization fit, quiet quitting, vocational college lecturers, work overload, work–family conflict

## Abstract

**Introduction:**

Quiet quitting has attracted growing attention as a form of work withdrawal, yet evidence from vocational education remains limited. Drawing on Conservation of Resources theory, this study examines how person–organization fit, work–family conflict, and work overload relate to quiet quitting among vocational college lecturers, with job burnout as a mediator.

**Methods:**

A cross-sectional online survey was conducted among vocational college lecturers in China, and 523 valid responses were analyzed using partial least squares structural equation modeling.

**Results:**

Person–organization fit negatively predicted quiet quitting and job burnout, whereas work–family conflict positively predicted both outcomes. Work overload did not directly predict quiet quitting but showed an indirect effect through job burnout. Job burnout significantly predicted quiet quitting and mediated the relationships between the three antecedent variables and quiet quitting.

**Discussion:**

These findings extend Conservation of Resources theory by showing that quiet quitting among vocational college lecturers is shaped by multi-level stressors and operates largely through job burnout, highlighting the importance of improving person–organization fit, reducing work–family conflict, and managing workload to sustain lecturer engagement.

## 1 Introduction

Quiet quitting is becoming an increasingly studied phenomenon in academia, as it represents a type of withdrawal from work that occurs when workers remain employed but choose to work only as to fulfill the most basic expectations of their job. Quiet quitting does not result in employees leaving an organization like voluntary turnover. Rather, it reflects a change in behavior such that employees decrease the amount of discretionary effort, emotional commitment, and extra-role behavior they exhibit (Aydin and Azizoğlu, 2022; Mahand and Caldwell, 2023). Quiet quitting represents a significant challenge for employers as, for the most part, there is no indication that someone is experiencing quiet quitting. While there are a number of reasons for this, one of the primary reasons is that it’s often difficult for employers to determine if someone is quit or not. Therefore, quiet quitting has a tremendous impact on both the effectiveness of the organization and the well-being of employees (Bulut et al., 2024; Serenko, 2024).

According to the science of behavior, the term “quiet quitting” is best categorized as a type of self-regulated behavior that can result from prolonged psychological strain. Rather than being interpreted as a transient change in attitude or lack of motivation, quiet quitting reflects a purposeful change of observable work behaviors (i.e., work behaviors) in response to long-term psychological strain (Bernuzzi et al., 2025). Research indicates that when employees interpret a lack of balance between their job demands and resources available to them, they might choose to engage in withdrawal-oriented behaviors as a means to conserve their functioning (Formica and Sfodera, 2022; Serenko, 2024). However, the existing body of research on quiet quitting has focused on empirical research conducted in corporate and health settings, while the majority of research around quiet quitting in education remains underexamined (Galanis, 2023; Pevec, 2023). This gap in research is critical, as working in educational institutions, particularly in vocationally oriented settings, is high in terms of emotional demands and usage of resources, therefore putting teachers at risk for withdrawal through behavior as a result of the stresses of their work.

The educational system is an important place to study the phenomenon of quiet quitting due to the reliance on emotional labor, commitment to one’s profession, and ongoing interpersonal relationship in teaching, putting teachers at risk for depletion of resources (Memiş and Tabancalı, 2024; Palad, 2023). Some teachers in all sectors of education have been under increased strain from increasing workloads and expectations for performance as well as tension between professional and personal life, causing teachers to be concerned about burnout, withdrawal from the job, and the decline in quality of service they provide. Despite there being a lot of research on burnout and withdrawal from job effects, the specific behaviors that link these stressors to quiet quitting in a teacher have not been adequately examined (Khan et al., 2025; Nazaruk et al., 2025).

Due to the particular nature of the vocational education system, the vocational college lecturer is subjected to an extremely rigorous work environment. Vocational college lecturers have additional responsibilities beyond their teaching duties (Wenya and Mohd, 2025). Their additional responsibilities include providing skills-based practical training to students, supervising them in the workplace, coordinating administrative tasks, and collaborating with external industry representatives (Zhao et al., 2023). As a result, they balance these two positions with high cognitive and emotional pressures on an ongoing basis. Furthermore, vocational college lecturers frequently experience significant levels of workload associated with administrative tasks, multiple-role performance assessment systems, and limited opportunities for promotion and advancement (Mohd Rokeman and Che Kob, 2024).

According to the Conservation of Resources (COR) Theory, multiple aspects of individuals can be interpreted as being in a state of continual and multi-dimensional resource depletion. The competing demands on time, energy and emotions created by teaching, administration and industry interaction cause rapid depletion of all three types of resources (Liangyu et al., 2024). There is little institutional support for resource replenishment or movement along career paths, thus limiting the opportunity to replenish or acquire new resources. Consequently, vocational college teachers may not have the option of leaving their organization due to structural and financial barriers. Therefore, vocational college instructors often see quiet quitting as a rational and adaptive means of conserving their remaining resources (Pharamela and Singh-Pillay, 2025). Unlike employees working in corporate and healthcare environments, where there may be mobility between jobs, role flexibility or the potential for an exit or role adjustment through an external source, vocational college instructors often respond to extended periods of stress by reducing the discretionary effort they exert while maintaining their adherence to their formal job responsibilities (Munyaradzi et al., 2024). Quiet quitting, therefore, is a behaviorally relevant response that is influenced by the structural constraints on available resources, rather than simply attitude or motivation.

The study uses Conservation of Resources theory to examine why vocational college faculty may engage in quiet quitting. Conservation of Resources theory argues that people will seek to obtain, maintain, and conserve their valued resources (i.e., time, energy, emotional capacity, and sense of meaning), and that stress is caused by the threat of being resource depleted or having lost resources over time, and that the prolonged loss of resources leads individuals to use defensive strategies in order to conserve their remaining resources (Hobfoll, 2011; Hobfoll et al., 2018). In this context, job burnout is viewed as a key psychological outcome of cumulative loss of resources and as a proximal mechanism linking workplace stressors to withdrawal behaviors.

The study extends the Conservation of Resources theory to hypothesize that multiple sources of resource strain contribute to quiet quitting of vocational college faculty, including P–O fit between the individual and the organization, contextual (WFC) and inter-role (WFC) factors, including work overload (Basha and Pathania, 2025; Galanis et al., 2024). Misalignment of employee values and organizational expectations (P–O Fit), interference with work and family roles (WFC), and excessive job demands (work overload) are expected to contribute to job burnout and, as a result, to quiet quitting (Mingming et al., 2024; Prentice et al., 2025; Rotenstein et al., 2023). While prior research has examined these factors independently, few studies have integrated them into a unified analytical framework to explain quiet quitting behavior in educational contexts.

The present research draws from the Conservation of Resources (COR) Theory and takes a multi-level view of the selected focus stressors, capturing varying sources of resource loss. The first focus stressor is Person–Organization Fit (P-O Fit). This is a form of personal resource aligning/connecting one’s personal values to the work environment where they are performing their job. The second focus stressor is the work–family conflict (WFC), which represents the stressors that come from the interaction between two contexts (work and family). The last focus stressor is work overload, representing an organizational level of demand based on the intensity of the tasks and the expectations of the employee’s role. All three of these constructs are included in this study because they complement and are also distinct, they represent different ways through which employees gain and lose resources at different levels throughout their work experiences. Other established predictors of stress (e.g., role conflict, emotional labor, job autonomy) are included to a lesser degree because those predictors are less relevant to vocational education college lecturers due to the nature of their jobs, such as alignment with the values of the institution’s mission (P − O Fit), boundary-spanning between work and personal life (WFC), and an existence of high task intensity levels (Kana and Letaba, 2024). Combined, the three chosen focus stressors represent an overarching view of the dynamics of resource loss through multiple sources that impact the behaviors of employees in quiet quitting.

It is noteworthy that COR theory recognizes the difference between resource generating conditions and resource depleting demands and therefore assists in explaining the differing roles of study variables (Demerouti, 2025). P–O Fit is considered a resource enabling condition which assists employees in acquiring and protecting resources through congruency between individual value systems and organization support systems (Zeng and Hu, 2024). This congruence provides employees with an increased sense of psychological safety, meaning, and energy thus reducing the need for defensive withdrawal behaviors (e.g., quiet quitting) as a direct result (Chen et al., 2024). Conversely, WFC and work overload are resource depleting stressors that continually consume an individual’s time, energy, and emotional resources (Tavassoli and Sunyer, 2025). However, these stressors may not produce a withdrawal behavior unless there is a threshold point reached whereby resource depletion is substantial enough such that there is an actual behavioral withdrawal. Within this context, job burnout is representative of a critical threshold reflecting a high level of resource depletion. Therefore, while P–O Fit is likely to influence silent quitting both directly and indirectly, WFC and work overload will likely only have an indirect influence via progressively depleting resources to induce burnout. This distinction provides a theoretical basis from which to understand the differing pathways tested in this study.

The further application of the conservation of resources (COR) theory highlights the loss spiral theory that the initial loss of resources creates an increase in chance of future loss, and therefore increases vulnerability to future loss. Indeed, individuals faces experience an increased amount of resource loss over time, they can likely develop defensive strategies to conserve what resources remain (Pham et al., 2024). Quiet quitting is perceived as a defensive withdrawal behavior (i.e., intentionally withdrawing discretionary effort and/or engagement) to prevent the loss of additional resources (Vyzul Karnine et al., 2024). From this viewpoint, quiet quitting is not necessarily disengagement from work, but rather viewed as a rational coping mechanism in a larger context of lost resources.

In particular, the purpose of this study is to examine how person–organization (P–O) fit and work–family conflict (WFC) influence quiet quitting behavior among vocational college lecturers through job burnout as a mediator. The study is designed using survey-based research from the vocational colleges in China in order to extend the application of COR theory to the field of vocational education. Additionally, this research contributes to behavioral research by identifying various pathways by which multiple types of stressors may lead to different behaviors related to work withdrawal. Finally, by identifying the psychological processes associated with quiet quitting behavior, this research also provides insights into how to mitigate job burnout and promote continued engagement through high demand educational environments.

## 2 Literature review

### 2.1 Person–organization fit and quiet quitting

P-O fit equals how aligned an individual’s values, expectations, and goals are with the values, expectations and goals of the organization (Kristof-Brown et al., 2023). It is a powerful psychological resource with which can affect the level of employee engagement and subsequent patterns of behavior (i.e., withdrawal-related). Evidence shows that employees who perceive a good P-O fit will have higher work motivation, lower stress levels and lower levels of withdrawal-related behaviors; however, if an employee perceives a lack of P-O fit will have lower enthusiasm at work and a higher likelihood of quitting quietly (Coskun et al., 2022; Mostafa et al., 2023; Nordgren and Björs, 2023).

Members of vocational colleges whose teaching practice, values of the organization and individual professional identity are strongly related to one another (Rasmet et al., 2025). P-O fit facilitates lecturers’ identification with their organizations, leading to greater levels of sustained engagement with their work, which will result in less likelihood of the member quitting quietly (Udin, 2024). Therefore, we propose the following hypothesis:

*H*_1_. P–O Fit has a negative impact on quiet quitting among vocational college lecturers.

### 2.2 Work–family conflict and quiet quitting

Work–family conflict (WFC) is defined by Greenhaus and Beutell (1985) as a type of inter-role conflict, in which the demands from both the work and family domains are incompatible. Following the COR framework, WFC is considered a prominent opportunity for resource loss, as competing demands of the roles continuously consume time, energy, and emotional resources resulting in increased strain and decreased work-related engagement. Studies have demonstrated that higher levels of WFC are related to lower engagement, higher emotional exhaustion, and greater wish to withdraw from work, all of which are strong predictors of quiet quitting (Nordgren and Björs, 2023; Ribeiro et al., 2023).

Within vocational colleges, lecturers often have to balance work-related (intensive workload and administrative responsibilities) and family-related obligations (Bahri et al., 2025). To conserve their remaining resources once WFC becomes overwhelming, lecturers begin to withdraw from their discretionary efforts and initiative, thereby becoming more likely to engage in quiet quitting (Lukmanul Hakim et al., 2025). Consequently, the following hypothesis is suggested:

*H*_2_. WFC has a positive impact on quiet quitting among vocational college lecturers.

### 2.3 Work overload and quiet quitting

Work overload refers to a condition in which employees perceive that their work demands exceed their available time, energy, or personal capacity. Within COR theory, work overload may accelerate resource depletion and weakens individuals’ ability to sustain high levels of work engagement. Prior research consistently shows that excessive workload is associated with reduced engagement, lower job satisfaction, and diminished discretionary effort (Moreno-Martínez and Sánchez-Martínez, 2025). In addition, empirical evidence indicates that heavy teaching, research, and administrative responsibilities significantly undermine lecturers’ performance and participation by increasing psychological strain and fatigue (Amie-Ogan and Fekarurhobo, 2021).

In vocational colleges, lecturers often face the overlap of multiple work tasks, which exposes them to sustained workload pressure (Manditereza and Chamboko-Mpotaringa, 2024). When such demands continuously exceed their coping capacity, lecturers may conserve remaining resources by reducing initiative, and lowering involvement in non-essential tasks, thereby increasing the likelihood of quiet quitting (Che Sobry et al., 2024). Based on these relationships, the following hypothesis is proposed:

*H*_3_. Work overload has a positive impact on quiet quitting among vocational college lecturers.

### 2.4 Person–organization fit, work–family conflict, work overload, and job burnout

Job burnout occurs when resources have been depleted over an extended period of time. Job burnout has three main symptoms: emotional exhaustion, depersonalization, and reduced professional competence (Maslach and Leiter, 2016). Recent studies consistently show that P-O Fit, WFC, and work overload are three of the most important antecedents to experiencing burnout on the job. When employees have a high P-O Fit with their company, they are able to experience lower levels of burnout. Specifically, having an employee’s values aligned with the organizational culture of a company increases an employee’s ability to be psychologically resilient and decreases their level of emotional strain (Liu and Xie, 2024). Conversely, WFC is a source of chronic role stress that negatively impacts an individual’s well-being and increases the level of emotional exhaustion (Zábrodská et al., 2018), while work overload will deplete both physical and psychological resources and subsequently lead to increased levels of role stress, thereby increasing the likelihood of developing burnout (Nickum and Desrumaux, 2023).

At vocational colleges, instructors typically have a variety of responsibilities involving teaching, conducting research, and providing practical training (Mazibuko and Maharaj, 2024). Therefore, they are especially vulnerable to being depleted of resources, given that they typically experience P-O fit, role conflict, and work overload. Given this predicament, the following two relevant hypotheses (grounded in COR Theory) are proposed:

*H*_4_. P–O fit has a negative impact on job burnout among vocational college lecturers.

*H*_5_. WFC has a positive impact on job burnout among vocational college lecturers.

*H*_6_. Work overload has a positive impact on job burnout among vocational college lecturers.

### 2.5 Job burnout and quiet quitting

Job burnout has been frequently noted as the primary source of withdrawal behavior (Memiş and Tabancalı, 2024). Burnout acts as an advanced level of resource depletion, according to the Conservation of Resources (COR) framework. Therefore, it is an important contributor to the way employees withdraw (e.g., disengaged, decreased effort, and poor role performance) (which are examples of quiet quitting; Hamouche et al., 2023; Serenko, 2024). Thus, employees who experience high levels of burnout will attempt to conserve available resources by minimizing their engagement, and avoiding new responsibilities.

In the educational context, there is also empirical evidence to support this relationship. For example, lecturers who are experiencing extended periods of job burnout may use less discretionary effort as employees remain employed in their positions and may also choose to quiet quit (Harmse and Dichaba, 2025). Therefore, the following hypothesis is offered:

*H*_7_. Job burnout has a positive impact on quiet quitting among vocational college lecturers.

### 2.6 The mediating role of job burnout

The available evidence supports that there are three major influences on the phenomenon of quiet quitting: P–O Fit, work–family conflict (WFC), and work overload, using the Conservation of Resources (COR) theory to explain the mechanisms through which they interact as resources. The P–O Fit generates resources for the psychological alignment of the employee and the organization creating a perception of support and availability of resources (Insan et al., 2025). Thus, P–O fit decreases the odds of engaging in withdrawal behaviors and lowers the chances of experiencing burnout. In contrast to P–O fit, WFC and work overload are considered resource-depleting stressors that deplete the employee’s cognitive, emotional, and temporal resources (Magagula and Awodiji, 2024). The impact of these stressors on behavioral outcomes is through an indirect effect, according to COR theory, because when individuals experience a resource loss, they first engage in attempts to manage and cope with that loss through employment behaviors, which are negatively impacted by experiencing withdrawal behaviors (Fullard, 2025). It is not until there is a significant and ongoing depletion of resources (through WFC or work overload) that the experience of job burnout will result. Together with this, job burnout will result in the employee engaging in defensive behaviors such as quiet quitting (Nagler et al., 2020).

In vocational colleges, multiple role pressures may therefore influence quiet quitting primarily through their impact on lecturers’ burnout. Therefore, we will test the following hypothesis:

*H*_8_. Job burnout mediates the relationship between P–O Fit and quiet quitting among vocational college lecturers.

*H*_9_. Job burnout mediates the relationship between WFC and quiet quitting among vocational college lecturers.

*H*_10_. Job burnout mediates the relationship between work overload and quiet quitting among vocational college lecturers.

**Figure 1:**
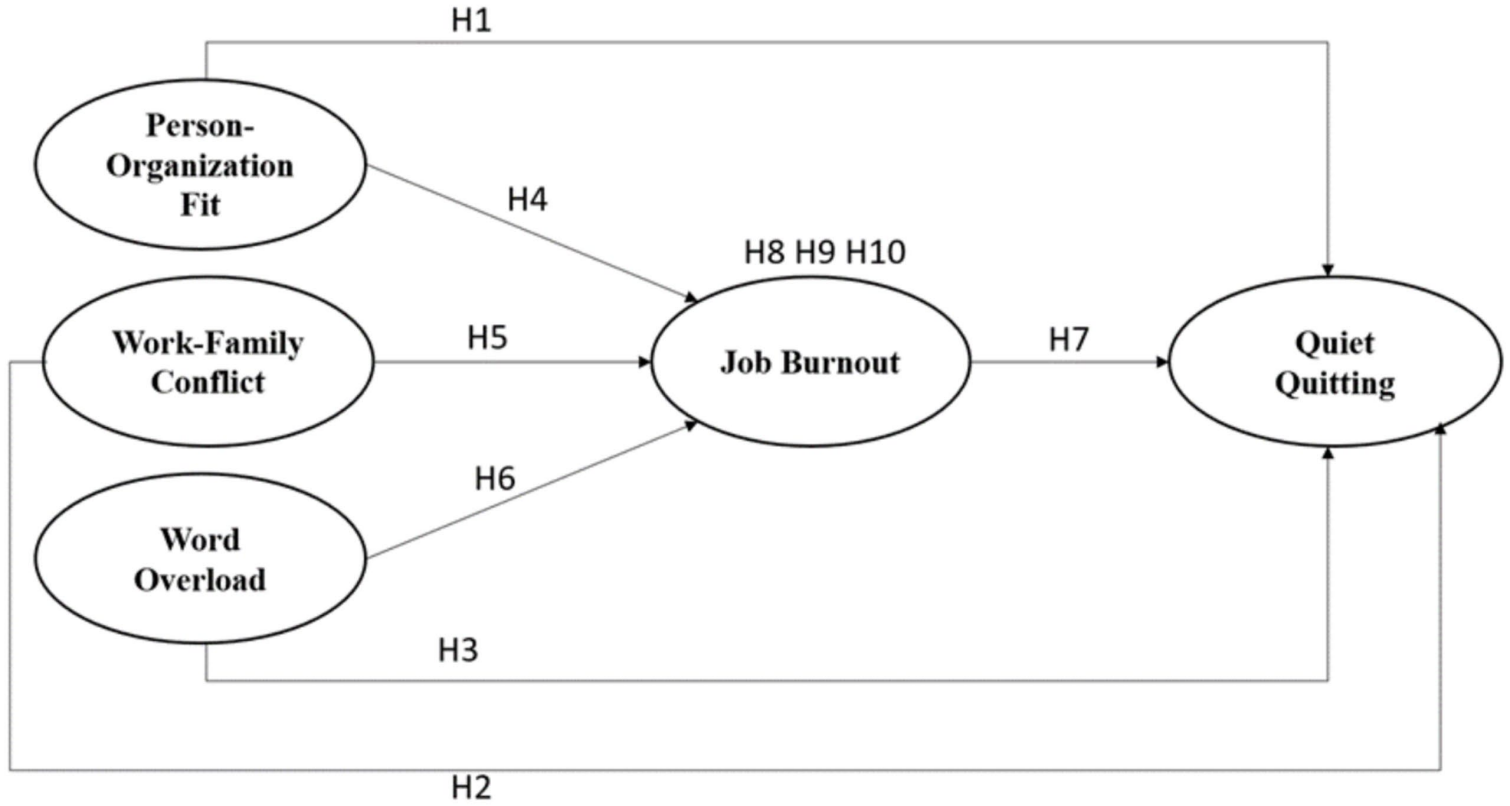
Research model.

The relationships based on the hypotheses are drawn in the model of this study, shown in Figure 1.

## 3 Research methods

### 3.1 Research sample and data sources

This study employed a randomized sampling approach to collect data from vocational college lecturers in China. To obtain a sufficiently representative and diverse sample of vocational colleges, we drew a random sample of 10 vocational colleges, representing the eastern, central, and western regions of China. Before conducting the formal survey, we performed a pilot study to refine the questionnaire, including the adapted quiet quitting scale items, in order to determine their clarity, contextual relevance, and content validity for the vocational college lecturers included in the study. The pilot study produced an initial sample of 61 lecturers, but subsequent analysis of the pilot study data resulted in modifications to the data set based on issues encountered during the administration of the pilot study. Using Krejcie and Morgan (1970) as a reference for determining a sample size, we determined that for sample sizes greater than 10,000 we needed a minimum of 384 responses. As part of the Teacher Development Centers at the selected colleges, we administered the formal survey through the Questionnaire Star platform in addition to utilizing various modes of institutional communication such as WeChat groups, QQ groups, and email. Data collection took approximately two (2) months, and we received a total of 588 responses to the survey. After performing data screening procedures, we excluded 65 responses due to outlier status or evidence of abnormal response patterns from our analysis, leaving 523 responses that were included for subsequent analysis. This final sample size exceeded the recommended minimum requirement and was considered adequate for statistical analysis.

There is a significant discrepancy between the sample distributions. More participants came from eastern China than central or western regions. The eastern regions have many more vocational schools, which are more accessible because of the regions’ higher economic development levels. However, there are many factors that could affect the stress and burnout levels registered by lecturers, including the resource allocation to institutions, the administrative policies that apply to the institutions, and the workload structures of their regions. Therefore, when interpreting the generalizability of the findings, we need to take the sample distribution into account.

The present research utilized a cross-sectional research design. All the variables were collected at one point in time. While this is a suitable design to examine the relationships between variables and test mediation through a structural equation model, it imposes limits on the validity of causation that can be established among constructs. For example, although job burnout and quiet quitting are based on COR theory, we cannot provide empirical evidence of the timing of when these constructs influence each other. As such, the research findings should be interpreted as relational in nature, rather than having an established, causal relationship.

### 3.2 Measurement tools

#### 3.2.1 Person-organization fit scale

The research conducted in this paper utilized the six-item scale used for measuring P–O Fit developed by Mahmut et al. (2015), which resulted from the theoretical work of Cable and Judge (1996); Kristof-Brown et al. (2005); and Saks and Ashforth (2002). The scale has shown high reliability (Cronbach’s *α* = 0.91) and structural validity in other literature and has been shown to have applicability within different organizational and cultural settings. The items reflect four main concepts of perceived fit: value congruence (i.e., self and job); job characteristics; interpersonal relationships; and organizational opportunities, making them suitable for assessing P–O Fit among vocational school instructors. All items were rated on a 5-point Likert scale, with a score of 1 indicating “strongly disagree” and a score of 5 indicating “strongly agree,” and higher scores reflecting greater levels of perceived P–O Fit (Appendix A).

#### 3.2.2 Work–family conflict scale

Using the Work–Family Conflict Scale, developed by Netemeyer et al. (1996) to assess WFC (Work–Family Conflict) with respect to both work interference with family (WIF) and family interference with work (FIW) was the approach used in this research study. The purpose of this research is to examine how work-focused demands result in resource depletion which leads to behavioral withdrawal; therefore, this research study focuses on the work interference with family (WIF) sub-scale of the Work–Family Conflict Scale to assess how work duties interfere with lecturers’ family obligations. According to COR (Conservation of Resources) theory, WIF represents a major pathway for potential resource loss for lecturers due to the temporal, physical, and emotional demands of work consuming time, effort, and emotional energy from all other areas. In vocational colleges, it appears that the pressure experienced while performing their teaching, administrative, and industry-related responsibilities is more likely to extend into their family life than the reverse. Therefore, WIF is considered more theoretically relevant to explaining resource depletion and quiet quitting behavior in this population. However, the exclusion of the family interference with work (FIW) dimension may limit the ability to capture the bidirectional nature of work–family conflict. Future research is encouraged to incorporate both WIF and FIW to provide a more comprehensive understanding of inter-role conflict dynamics. Prior research reported a Cronbach’s alpha value of 0.871 for these items, indicating good reliability (Su and Jiang, 2023).

It is also important to note that the use of the WIF sub-dimension reflects a construct-specific operationalization rather than a full representation of work–family conflict. While this approach is appropriate for capturing work-driven resource depletion in line with COR theory, it may not fully account for the bidirectional nature of inter-role conflict. Therefore, the findings related to WFC should be interpreted within the scope of work-to-family interference, and future research is encouraged to incorporate both WIF and FIW to provide a more comprehensive assessment of work–family dynamics.

#### 3.2.3 Work overload scale

The Role Overload Scale (ROS) developed by Peterson et al. (1995) was used to measure work overload in this study. The ROS is a well-established measure of work-related role overload that is commonly used both in Chinese and international research settings. The ROS is based on the employee’s subjective perception of how much work he or she has to do, and it has shown to be reliable and applicable in many different types of work. The authors of this study adapted five of the items on the ROS to assess the perception of work overload on vocational college teachers. The five items were rated on a five-point Likert scale, giving the reader the ability to quantify their perception of work overload. The CRC has consistently reported acceptable internal consistency for this measure, (Cronbach’s *α* = 0.891), indicating that it is appropriate to use in this study (Li et al., 2022).

#### 3.2.4 Job burnout scale

The Maslach Burnout Inventory—General Survey was used to determine job burnout in the present sample. Burnout was conceptualized as a multidimensional construct including components of emotional exhaustion, cynicism, and lack of personal accomplishment. Based on the focus of this study being on Chinese recruits in vocational colleges as their career, Wu et al. (2021) developed a revision of the MBI-GS based on the Chinese culture that has been extensively researched to validate the Chinese context as an appropriate tool to measure teacher burnout for vocational college lecturers. Each of the 10 items of the MBI-GS were also rated using a 5 point scale, with higher scores reflecting higher levels of burnout in their job. Prior studies have reported that this scale has a very high internal reliability (Cronbach’s *α* = 0.915). This indicates that the MBI-GS was an appropriate measure for the current study (Jiang, 2018).

#### 3.2.5 Quiet quitting scale

The researchers used the Quiet Quitting Scale (QQS), developed by Anand et al. (2024), to measure the concept of ‘quiet quitting.’ This scale conceptualizes quiet quitting as a type of behavioral disengagement where an employee exerts little effort, demonstrates low commitment, and shows minimal to no discretionary involvement. The researchers adapted seven of the items from the QQS to suit the context of vocational college teaching. They made minor changes to the wording to better represent the academic setting (e.g., teaching task fulfillment, attendance to meetings, and engagement with the institution) while maintaining the original conceptual meaning. They ensured that the adapted items were relevant to the context and contained content validity by having multiple subject matter experts in organizational behavior and education review the adapted items and conducting a pilot study consisting of a small number of lecturers before the primary data collection. The researchers used the feedback received from the pilot study to improve item clarity and context validity. All items were measured on a five-point Likert-type scale with higher scores indicating higher levels of quiet quitting. The QQS demonstrated high internal consistency (Cronbach’s *α* = 0.829) indicating the reliability of the measure for the current study.

This research takes both internationally validated measures and measures tailored to the cultural context into consideration when deciding what measurement to use. For example, when measuring P–O Fit, work–family conflict, and work overload, the researcher used established, internationally recognized measures developed in Western countries. Past research has shown that these measures can also be built and adapted for use in organizations and educational institutions in China. Job burnout was measured using Li Chaoping’s adapted version of the Maslach Burnout Inventory-GS (MBI-GS), which has been developed for use within Chinese culture. This approach is designed to give a balance of theoretical consistency as well as culturally relevant measures. Future research should continue to validate these measures in vocational education settings or develop localized measures to improve cultural awareness and measurement equivalency.

### 3.3 Statistical analysis

Data was analyzed with SPSS 26.0 and Smart PLS 4.0. Descriptive statistics for response rates, demographic characteristics, and preliminary data screening were completed in SPSS 21.0 to check data quality and possible validity and reliability issues, prior to conducting Partial Least Squares Structural Equation Modeling (PLS-SEM) with Smart PLS 4.0. The measurement model was evaluated by determining the convergent and discriminate validity, then the structural model was evaluated for the hypothesized relationships between the selected variables. Bootstrapping procedures were used to assess the significance of direct/indirect (i.e., mediating) effects. In addition, the analysis of group differences across key demographic characteristics were assessed to provide evidence for further interpretation and discussion of the results.

## 4 Results

### 4.1 Descriptive statistics

The results presented in Table 1 indicate that a majority of the participants were female and represented 63.7% of all respondents while males accounted for 36.3%. Regarding marital status (married: 51.4%; single: 29.6%; other: 18.9%), the majority of participants possessed a master’s degree (64.4%), followed by bachelor’s (24.9%) and doctoral degrees (10.7%). The respondents’ years of teaching experience indicated that the largest category of lecturers had between 10 and 15 years of experience (31.7%), while those with 5 to 10 years comprised the second largest group (27.2%). Based on their professional rank, over 70% of the sample were senior or associate professors and only 9.9% reported having the title of professor. Geographically, the majority of respondents were from vocational colleges located in eastern China (67.5%); 15.3% were from central and 17.2% were from western China, which reflects the regional distribution of the population of lecturers at the privately-operating vocational colleges sampled.

**Table 1:** Sample demographics (*n* = 523).

Variables	Groups	Frequency distribution	Percentage (%)
Gender	Male	190	36.3
	Female	333	63.7
Marital status	Married	269	51.4
	Single	155	29.6
	Other	99	18.9
Educational Level	PhD (Doctorate)	56	10.7
	Master’s Degree	337	64.4
	Bachelor’s Degree	130	24.9
Years of teaching experience	Less than 5-year	84	16.1
	5–10 years	142	27.2
	10–15 years	166	31.7
	15–20 years	110	21.0
	20–25 years	21	4.0
Academic Titles	Lecturer	78	14.9
	Senior lecturer	160	40.3
	Associate professor	210	30.6
	Professor	52	9.9
	Other	23	4.4
The region where your vocational college is located	Eastern China	272	67.5
	Central China	117	15.3
	Western China	134	17.2

For the descriptive analysis, statistical procedures were performed with IBM SPSS 26.0 (see Table 2). The descriptive statistics presented for each measurement item ranged from a mean of 2.736 to a mean of 3.922, indicating moderate to moderately high levels of responses for all measured variables. The standard deviations ranged from 1.014 to 1.315 and therefore suggested an adequate amount of differences among lecturers for the individual measured variables. The assessment of the distribution characteristics for the data included evaluating the skewness and kurtosis values. Skewness ranged from −0.834 to 0.525 and kurtosis ranged from −1.013 to 0.401, all of which were within acceptable limits of approximate normality (Kline, 2023). The above results suggest that there were sufficient levels of dispersion and distributional characteristics in the data that permit subsequent analyses.

**Table 2:** Descriptive statistics for all measurement items.

Item	Mean	Standard deviation	Skewness	Kurtosis
POF1	3.883	1.088	−0.770	−0.166
POF2	3.922	0.977	−0.834	0.401
POF3	2.910	1.240	0.074	−0.872
POF4	3.120	1.079	0.044	−0.601
POF5	3.293	1.165	−0.214	−0.740
POF6	3.377	1.014	−0.309	−0.247
WFC1	3.107	1.133	−0.148	−0.639
WFC2	3.143	1.226	−0.081	−0.906
WFC3	3.164	1.171	−0.129	−0.791
WFC4	3.054	1.232	0.003	−0.980
WFC5	3.293	1.145	−0.260	−0.664
WFC6	3.010	1.222	0.020	−0.878
WO1	3.440	1.078	−0.539	−0.090
WO2	3.174	1.149	−0.139	−0.621
WO3	3.256	1.164	−0.130	−0.695
WO4	3.455	1.159	−0.468	−0.450
WO5	3.197	1.240	−0.124	−0.962
JB1	3.304	1.194	−0.184	−0.848
JB2	3.411	1.142	−0.396	−0.468
JB3	3.296	1.096	−0.292	−0.504
JB4	3.365	1.107	−0.597	−0.228
JB5	2.902	1.168	0.082	−0.848
JB6	2.922	1.116	0.122	−0.665
JB7	3.075	1.196	−0.002	−0.884
JB8	2.973	1.143	−0.009	−0.801
JB9	2.914	1.156	0.154	−0.783
JB10	3.080	1.170	−0.049	−0.789
QQ1	2.482	1.160	0.525	−0.599
QQ2	3.424	1.279	−0.424	−0.862
QQ3	2.929	1.315	0.106	−1.126
QQ4	3.052	1.261	−0.034	−1.013
QQ5	3.122	1.219	−0.159	−1.002
QQ6	2.799	1.208	0.214	−0.857
QQ7	2.736	1.255	0.240	−0.893

POF, Person-Organization Fit; WFC, Work–Family Conflict; WO, Work Overload; JB, Job Burnout; QQ, Quiet Quitting.

### 4.2 Measurement model assessment

**Figure 2:**
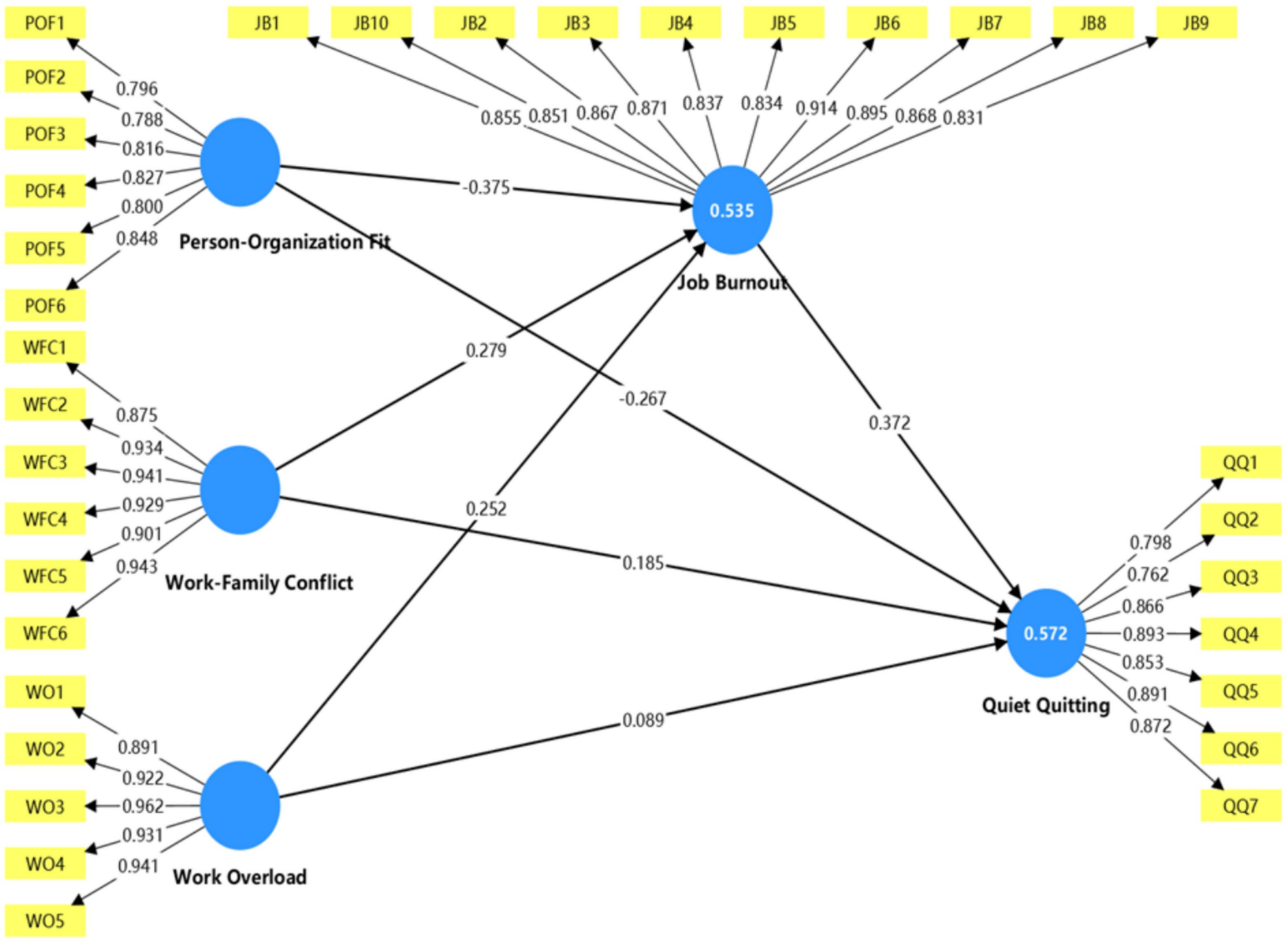
PLS-SEM measurement model.

The next step after completing the Descriptive Analysis was to assess the Measurement Model (illustrated in Figure 2). This was done to establish the Reliability and Validity of all the Constructs prior to performing Structural Model Tests. To evaluate Internal Consistency Reliability, both Cronbach’s Alpha and Composite Reliability (CR) were calculated. Table 3 provides the results of this evaluation with the Cronbach’s Alpha values ranging between 0.898 and 0.964, and the CR values ranging from 0.921 to 0.971. All of the Constructs have exceeded the recommended Threshold (0.70).

**Table 3:** Convergent validity analysis summary.

Indicators	Item	Factor loadings	Cronbach’s alpha	Composite reliability(CR)	Average variance extracted (AVE)
Job Burnout	JB1	0.855	0.962	0.967	0.744
	JB2	0.867			
	JB3	0.871			
	JB4	0.837			
	JB5	0.834			
	JB6	0.914			
	JB7	0.895			
	JB8	0.868			
	JB9	0.831			
	JB10	0.851			
P- O Fit	POF1	0.796	0.898	0.921	0.660
	POF2	0.788			
	POF3	0.816			
	POF4	0.827			
	POF5	0.800			
	POF6	0.848			
Quiet quitting	QQ1	0.798	0.935	0.947	0.721
	QQ2	0.762			
	QQ3	0.866			
	QQ4	0.893			
	QQ5	0.853			
	QQ6	0.891			
	QQ7	0.872			
WFC	WFC1	0.875	0.964	0.971	0.847
	WFC2	0.934			
	WFC3	0.941			
	WFC4	0.929			
	WFC5	0.901			
	WFC6	0.943			
Work overload	WO1	0.891	0.961	0.970	0.864
	WO2	0.922			
	WO3	0.962			
	WO4	0.931			
	WO5	0.941			

POF, Person-Organization Fit; WFC, Work–Family Conflict; WO, Work Overload; JB, Job Burnout; QQ, Quiet Quitting.

The assessment of Convergent Validity was achieved using the Standardized Factor Loadings (SFL) and Average Variance Extracted (AVE). All item SFL were above 0.708 and ranged from 0.788 to 0.962, while the AVE values ranged from 0.660 to 0.864. Therefore, all Constructs satisfied the requirement for adequate Convergent Validity (see Table 3).

To assess discriminant validity, both the Fornell-Larcker criterion and the Heterotrait-Monotrait ratio (HTMT) were used. The AVE’s (average variance extracted) square root for each construct was higher than the correlations between constructs (refer to Table 4) and all HTMT’s were below 0.90; with the highest HTMT of 0.725 (refer to Table 5). Therefore, there was satisfactory discriminant validity. Overall, the measurement model demonstrated acceptable reliability and validity which supports later conducting structural model analyses.

**Table 4:** Fornell-Larcker criterion.

Constructs	Job burnout	P–O fit	Quiet quitting	Work overload	WFC
Job burnout	0.863				
P–O fit	−0.583	0.813			
Quiet quitting	0.693	−0.594	0.849		
Work overload	0.581	−0.362	0.531	0.930	
WFC	0.611	−0.419	0.587	0.695	0.921

**Table 5:** Heterotrait-Monotrait ratio (HTMT).

Constructs	Job burnout	P–O fit	Quiet quitting	Work overload	WFC
Job burnout					
P–O fit	0.600				
Quiet quitting	0.725	0.626			
Work overload	0.600	0.365	0.550		
WFC	0.635	0.436	0.614	0.716	

### 4.3 Structural model assessment

**Figure 3:**
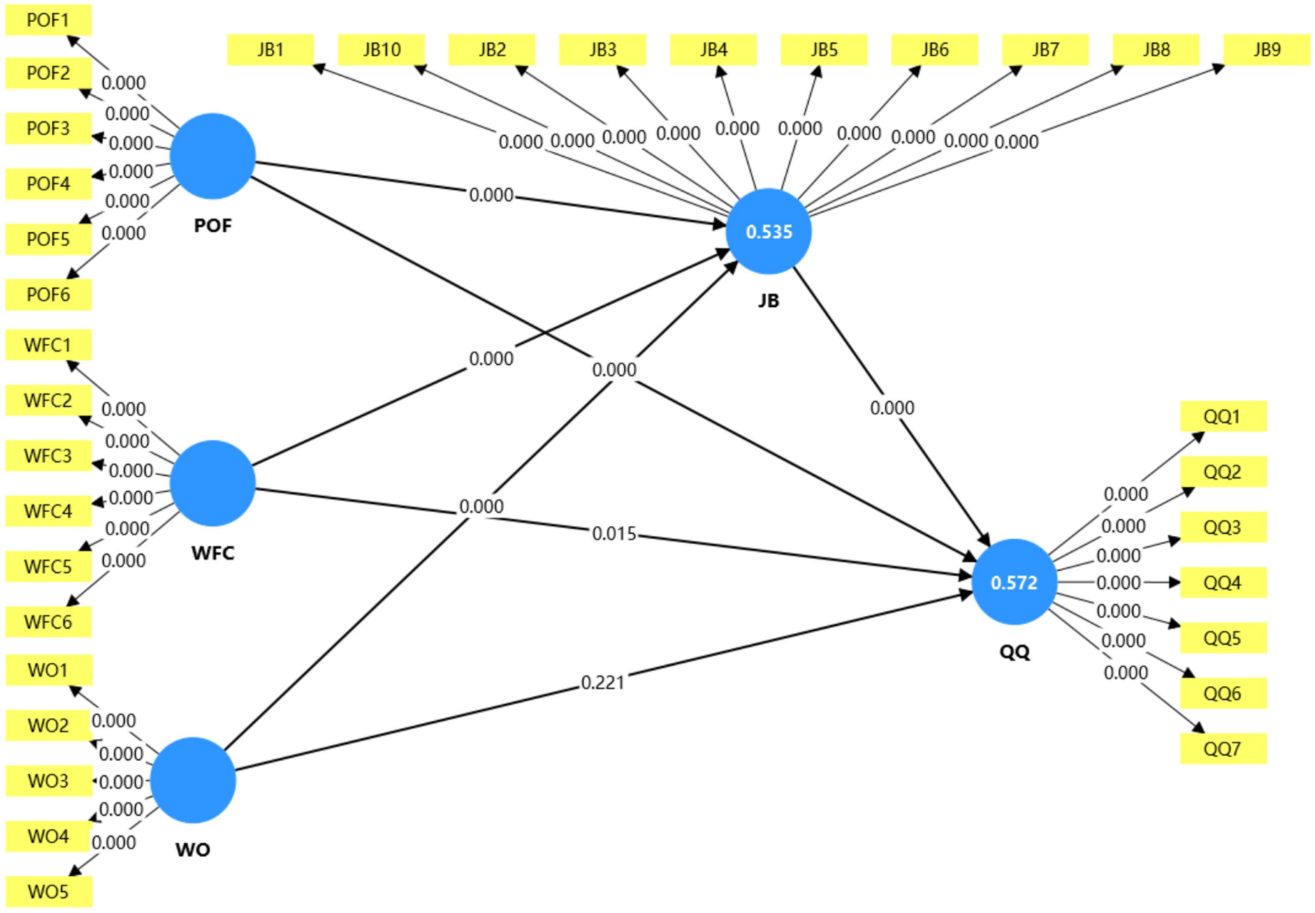
PLS-SEM structural model.

After establishing the reliability and validity of the measurement model, the structural model was evaluated using partial least squares structural equation modeling (PLS-SEM), as shown in Figure 3. A bootstrapping procedure with 5,000 resamples was applied to examine the significance of path coefficients and mediation effects.

The results of the path analysis are presented in Table 6. P–O Fit had a significant negative effect on quiet quitting (*β* = −0.267, *p* < 0.001), supporting H_1_. Whereas, WFC showed a significant positive relationship with quiet quitting (*β* = 0.185, *p* < 0.05), supporting H_2_. In contrast, the direct effect of work overload on quiet quitting was not significant (*β* = 0.089, *p* > 0.05), and thus H_3_ was not supported. Regarding job burnout, P–O Fit exerted a statistically significant negative effect (*β* = −0.375, *p* < 0.001), whereas both WFC (*β* = 0.279, *p* < 0.001) and work overload (*β* = 0.252, *p* < 0.001) showed significant positive effects, supporting H_4_–H_6_. Job burnout was found to have a strong positive effect on quiet quitting (*β* = 0.372, *p* < 0.001), confirming H_7_.

**Table 6:** Path coefficients and hypothesis testing.

Hypothesis	Path relationships	Path coefficient	Standard deviation	t-value	*p*-value
H_1_	P–O Fit→Quiet Quitting	−0.267	0.045	5.933	0.000
H_2_	WFC → Quiet Quitting	0.185	0.076	2.439	0.015
H_3_	Work Overload→ Quiet Quitting	0.089	0.073	1.224	0.221
H_4_	P–O Fit →Job Burnout	−0.375	0.044	8.595	0.000
H_5_	WFC → Job Burnout	0.279	0.060	4.659	0.000
H_6_	Work Overload→ Job Burnout	0.252	0.055	4.581	0.000
H_7_	Job Burnout → Quiet Quitting	0.372	0.076	4.901	0.000

The model’s explanatory power was measured by the coefficient of determination (R^2^). The P–O Fit, WFC, and Work Overload variables explained 53.5% of job burnout (R^2^ = 0.535), and the P–O Fit, WFC, Work Overload, and Job Burnout variables collectively explained 57.2% of quiet quitting (R2 = 0.572). The guidelines for PLS-SEM state that R^2^ values of 0.25, 0.5, and 0.75 are weak, moderate, and strong, respectively, according to Hair et al. (2019). Therefore, R2 values from this study are indicative of moderate to substantive explanatory power.

These findings are substantially above what could be found through prior studies using COR Theory and employee withdrawal behaviors, which confirms the moderate to strong explanatory power of the model in capturing a significant amount of variance across both outcomes (i.e., job burnout and quiet quitting) as a function of the complex nature of employee behaviors within organizations (Tan and Ismail, 2025). In turn, these relatively high levels of explanatory power demonstrate the predictive validity and robustness of the model proposed within the framework examined for vocational lecturers (see Table 7).

**Table 7:** Coefficient of determination (R^2^).

Variable	R-square
Job Burnout	0.535
Quiet Quitting	0.572

Using bootstrapping, mediation analysis was performed to determine whether job burnout can mediate the indirect effects of P–O Fit and WFC with respect to the outcomes of this study (quiet quitting). Table 8 presents the results of the mediation analysis, which found that job burnout does significantly mediate the relationship between P–O Fit and quiet quitting (*β* = −0.139, *p* < 0.001), providing support for H8. As the direct effect of P–O Fit on quiet quitting was also statistically significant (see Table 6), this indicates that the mediation effect is partial. Consequently, it is suggested that P–O Fit is a stable psychological resource and thus reduces quiet quitting directly; however, it also helps reduce quiet quitting indirectly by reducing job burnout. Job burnout also significantly mediated the relationship between WFC and quiet quitting (*β* = 0.104, *p* < 0.01) providing support for H9. As the direct effect of WFC on quiet quitting was statistically significant, this indicates that the mediation effect is partial. Thus, WFC contributes directly to behavioral withdrawal, but also indirectly intensifies it through resource depletion leading to burnout.

**Table 8:** Bootstrapping results of indirect effects.

Hypotheses	Path relationships	Path coefficient	Standard deviation	t-value	*p*-value	Lower	Upper
H_8_	P–O Fit →Job Burnout→ Quiet Quitting	−0.139	0.031	4.470	0.000	−0.210	−0.088
H_9_	WFC → Job Burnout→Quiet Quitting	0.104	0.036	2.880	0.004	0.050	0.189
H_10_	Work Overload→ Job Burnout → Quiet Quitting	0.094	0.032	2.972	0.003	0.045	0.167

Alternatively, job burnout was identified as completely mediating the path from excessive workload to quiet quitting (*β* = 0.094, *p* < 0.01), corroborating H10. To illustrate why this indicates complete mediation, the direct relationship from excessive workload to quiet quitting (see Table 6) did not reach statistical significance. Therefore, these results indicate that excessive workload does not result in a direct experience of withdrawal from the job but instead a gradual depletion of resources, eventually being manifested as job burnout, which subsequently leads to exhibiting behaviors associated with quiet quitting (e.g., reduced productivity).

## 5 Discussion

### 5.1 Quiet quitting as a resource-conservation response

Based on the Conservation of Resources (COR) perspective, quiet quitting is viewed as a way for vocational college instructors to preserve their scarce psychological resources (those they have already lost) while continuing to be formally employed (Wang and Hall, 2021). The results support this view by showing that quiet quitting is associated with two types of conditions: stable resource conditions (P–O Fit), and continuing resource stressors (WFC and work overload), as opposed to being temporary emotional responses or signs of lacking a work ethic.

It appears that lecturers modify their engagement in work when they perceive that they cannot recover lost resources. Under vocational education circumstances, where there are strong disincentives to leave, such as having a high degree of professional identity and institutional affiliation, as well as limited external employment opportunities, quiet quitting is a low-pressure and effective way for lecturers to withdraw from work (Zhou et al., 2025). The findings of the current study represent an enrichment of the existing literature (Martadiani et al., 2025), by providing empirical support for a framework of resource-based coping in relation to quiet quitting, as opposed to explanations related to generational differences or differences in motivation.

### 5.2 Differential effects of P–O fit, WFC, and work overload

The findings highlight several unique pathways to the influence of various stressors on quiet quitting. P-O fit has a significant and direct negative impact on quiet quitting, indicating its role as a stable psychological resource. When lecturers perceive a good fit with the values and practices of their organization, they will likely not disengage from their work even when subjected to demanding working conditions (Mat Zaki et al., 2024). This supports the argument that having like values can have long-term protective benefits against withdrawing from your workplace by helping to create a sense of purpose in the roles, creating a sense of identity within the academic community, and providing emotional security (Donald et al., 2025).

On the other hand, WFC has a significant positive impact on quiet quitting. Continuous interference across work and family roles leads to the natural depletion of resources, to which lecturers respond by reducing their discretionary effort as a self-preservation strategy in times of increasing demands (Hossain et al., 2012). This emphasizes the influence that strain caused by contextual role conflict has on withdrawal behavior, especially in professions with a high degree of borderless and ambiguous work-life boundaries.

Notably, work overload does not directly predict quiet quitting. This finding is theoretically significant and can be better understood within the context of vocational education. Prior research suggests that heavy workloads are often normalized among vocational college lecturers due to the inherent structure of their roles, which combine teaching, administrative responsibilities, and industry-related tasks (Perveen et al., 2025). As a result, lecturers may perceive high workload not as an exceptional stressor, but as a routine aspect of their professional responsibilities.

From a COR theory perspective, this normalization implies that workload pressure alone may not immediately trigger behavioral withdrawal. Instead, lecturers initially attempt to cope with increased demands by investing additional effort and resources (Usman Saleh et al., 2025). However, when such demands persist and exceed their capacity for recovery, cumulative resource depletion associated with job burnout. It is at this stage when psychological exhaustion becomes pronounced that lecturers begin to conserve remaining resources by reducing discretionary effort, resulting in quiet quitting (Mogashoa and Makaring, 2024). This explains why work overload does not exhibit a significant direct effect on quiet quitting but exerts its influence indirectly through burnout. This study provides a more precise perspective on existing knowledge in regards to workload-induced stress and withdrawal behaviors by identifying that there is not an automatic behavior to withdraw from job-related duties that occur when someone has experienced a high level of work-related stress, but that these behaviors are dependent on whether or not the individual has absorbed that stress as psychological fatigue (burnout).

The application of COR theory to this distinction comes from differentiating between resource generating and resource depleting behaviors. P–O Fit as a strong and durable resource will enhance engagement through its direct support of effort and energy, whereas WFC and work overload will drain that energy through continuous depletion of each resource until they reach a point of cumulative depletion (a critical level) and trigger burnout (a psychological state indicative of withdrawal). Thus, workload will not directly predict ‘quiet quitting’, but instead has an indirect effect on this behavior via burnout.

Findings from this research suggest that the strength of the negative relationship between P-O Fit and quiet quitting found in this study is stronger than what is typically reported in corporate contexts. In employment settings where there is a high rate of job mobility, employees who experience misfit are likely to leave the position rather than attempt to change the way they behave in the workplace (Donald et al., 2025). However, because vocational college lecturers work in a low job mobility environment with a high level of attachment to their institution, P-O Fit is an even more important psychological resource for employees than it would be for employees working in employment situations with a relatively high rate of job mobility (Harmse and Dichaba, 2025). Consistent with research conducted in educational settings regarding alignment with institutional values being an important aspect of teacher commitment, this study extends previous research by providing empirical evidence that alignment with institutional values has direct implications for the behavior of employees who are less likely to quit quietly than employees who do not have a high level of P-O Fit.

### 5.3 Job burnout as the proximal mechanism

Quiet quitting can be exacerbated by job burnout (Guan and Jamil, 2025). According to COR theory, burnout is a heightened level of resource depletion typically accompanied by emotional exhaustion and a decrease in professional effectiveness. When lecturers reach this point, they may limit how much they engage with students, how much initiative they take, and how many extra-role behaviors they perform as they try to conserve the resources that they still have available to them (Wang et al., 2022).

Thus, while lecturers may still carry out their regular duties as teachers, job burnout decreases their initiative and their willingness to engage voluntarily with their students. As a result, they may quietly quit instead of formally leaving (Ilham and Olle, 2024). This is especially true in vocational education, where job stability and ethical behavior can make it difficult for lecturers to resign from their jobs.

The findings from previous studies on educational institutions such as K–12 school teachers and university teachers show a high correlation between burnout and withdrawal behaviors, reduced quality of instruction, and disengagement at work (Kim et al., 2025). However, since there are many structural and organizational constraints on the expression of burnout for vocational college teachers compared to the previous contexts, quiet quitting occurs as opposed to turnover or absenteeism (Kim and Maijan, 2024). This illustrates that while the relationship between burnout and withdrawal is consistent across educational institutions, the behavioral manifestation of this relationship may vary according to the flexibility an employee has at work and their ability to leave.

### 5.4 Job burnout as a mediating threshold

Mediation analysis indicates that job burnout is a significant link between stressors and quiet quitting. Burnout clearly mediates the effect of work overload and partially mediates the effects of person–organization (P–O) fit and work–family conflict (WFC) on quiet quitting, illustrating that the way stressors cause quiet quitting depends on the cumulative loss of resources resulting in burnout.

The lack of a statistically significant direct effect of work overload but a significant indirect effect through burnout provides a critical theoretical insight. This suggests that workload is an externally-imposed stressor and that the external stressor has no impact on behavior until psychological exhaustion is incurred (Marliana Mohamad et al., 2025). This finding further defines stress–outcome models by positioning burnout as a necessary condition for the quiet quitting that occurs due to workload (Bahagia et al., 2024).

In vocational education settings, the high workload is integrated into the position of vocational educator and sustained for long periods of time (Mat Zaki et al., 2024). Thus, the results of this study indicate that it is not merely the voluminous amount of workload placed upon vocational educators that leads them to engage in a type of “quiet quitting,” but more importantly, it is their inability to recover from the prolonged demands of employment duties that influences how much vocational educators will continue to obtain employment as qualified vocational educators (Magagula and Awodiji, 2024). The nature of this distinction illustrates a need for differentiation between the external job demands placed on vocational educators, and the internal psychological conditions experienced by vocational educators, in explaining withdrawing behavior.

### 5.5 Theoretical implications

This research makes several contributions to the literature on quiet quitting and COR theory. One contribution is that it extends COR theory’s explanation of implicit behavioral withdrawal in education through the conceptualization of quiet quitting as a resource-conservation reaction to work-related stress rather than an attitudinal, or modern generational, phenomenon. Findings of the current research indicate that, because resource availability has been exhausted, professors reduce their efforts while remaining formally employed; thus, quiet quitting cannot be compared with overt turnover.

Secondly, this research advances theory by proposing and validating a multi-source stress–burnout–withdrawal model. The proposed model identifies how the accumulation of individual P-O Fit (i.e., resources); work–family conflict (WFC) (i.e., contextual stressor); and work overload (i.e., organizational demand) lead to the long-term, continual accumulation of resource-related pressures that manifest as quiet quitting via job burnout. These findings support the need for theoretical explanations of the phenomenon of quiet quitting.

One major theoretical contribution of the study is its finding that the direct, non-significant effect of work overload on quiet quitting is completely mediated by job burnout; which is contrary to the assumptions that an excessive workload causes an employee to withdraw from work and indicates that behavioral withdrawal occurs primarily when the emotional strain of meeting work demands becomes too much for someone (this is exemplified through psychological burnout). This clarification helps refine the COR theory by identifying how job-related demands can lead to job-related behaviors.

Finally, the researchers extended their research regarding quiet quitting beyond the corporate and university settings to include vocational college teachers and, in doing so: develop their theoretical frameworks and components more fully; within educational institutions.

### 5.6 Practical implications

The results of this investigation have some specific implications for administrators and policymakers in vocational post-secondary institutions. First, to promote enhanced person–organization fit (P–O Fit), appropriate HR practices need to be implemented in a systematic manner. For example, when recruiting new lecturers, vocational colleges should clearly articulate the values and priorities of the institution (e.g., integrating with industry, applied teaching and developing skills for students) during the recruitment and selection process via the recruitment process using values-based recruitment. New lecturer counts need to be aligned with institutional expectations via providing structured onboarding programs. Participatory governance mechanisms can enhance the alignment of values between the institution and new lecturers by allowing lecturers to be involved in curriculum development, planning with industry for collaboration, and participating in academic decision-making. Creating open and transparent systems for promotions and evaluations that recognize both teaching and industry engagement will foster greater organizational fit.

Second, to reduce work–family conflict (WFC), vocational colleges can help facilitate institutional adjustment targeted to the specific needs of lecturers. For example, vocational colleges can help reduce role conflict and uncertainty by providing flexible scheduling policies (e.g., planning teaching schedules and industry placements in advance). The administrative workload can be streamlined by reducing duplication in reporting, and by digitizing routine procedures. Additionally, institutions can provide support for lecturers to manage competing demands for their roles by implementing family-friendly leave policies or workload modifications during periods of acute family need.

To effectively address work overload from teaching, lecturing, and responding to student demands, colleges must implement structured workload management systems. A formal mechanism to monitor workloads needs to be developed to assess regularly the distribution of teaching hours, administrative responsibilities and any other industry-related tasks. Colleges can develop very clear guidelines for workload allocations, creating an equitable distribution of responsibilities among lecturers. Another way to reduce the level of workload pressure on lecturers would be to provide administrative assistants/support staff to perform non-academic tasks for lecturers. Additionally, implementing early warning systems (such as periodic assessments of burnout or well-being) can be beneficial to detect at-risk lecturers and intervene in a timely manner prior to resource depletion leading to disengagement.

These findings indicate that effective management of quiet quitting within the vocational education sector requires moving from broad performance-based expectations to developing resource-based targeted interventions that incorporate alignment, flexibility, and sustainable design of the lecturer’s workload.

## 6 Conclusion

This research used the Conservation Of Resources (COR) theory to evaluate how the Fit between the Person and Organization (P–O Fit), Work–Family Conflict (WFC) and Work Overload relate to quiet quitting behaviors of lecturers in Chinese Vocational Colleges. The analysis of the data using the Partial Least Squares Structural Equation Modeling (PLS-SEM) technique revealed that quiet quitting is indicative of a series of systematic responses to individual depletion of resources (and not merely a random change in attitude toward work). P–O Fit acted as a mediating variable between the dependent variable (quiet quitting) and independent variable (job burnout) is helpful in the prediction of job burnout. Additionally, WFC has a positive impact on both job burnout and quiet quitting. Work overload was found to not have a direct effect on quiet quitting, however, it influenced quiet quitting through the mediated pathway of job burnout to quiet quitting, indicating that job burnout serves as the critical threshold of transforming job-related stress into withdrawal form of behavior. This research also adds to the researchers’ understanding of quiet quitting as a type of hidden withdrawal and extends the COR theory by outlining how employees engaged in vocational education conserve the resources they have available to them while actively maintaining their employment with the organization. Finally, there are some limitations in this research pertaining to using a cross-sectional design that limits the researchers’ ability to infer causality as well as the researchers’ ability to ascertain the temporal ordering of the variables included in the research. Future research could adopt longitudinal designs and examine moderating factors such as organizational support or occupational identity to further refine the proposed model.

The regional make up of our sample is an important factor when considering the generalizability of the results. Most of the respondents were from vocational colleges located in the coastal regions of Eastern China. Other factors such as the relative level of development of these institutions, their relative level of available resources, and the administrative and performance expectations they face differ significantly from vocational institutions in Central and Western China. Given the different contextual factors, this may impact the work intensity experienced by faculties and level of institutional assistance available to faculties as well as how faculty cope with work-related stressors. From a COR perspective, differences in the context such as institution resource availability can impact both the acquisition and depletion of resources and therefore potentially lead to different levels of burnout or quiet quitting among faculties. Hence, the findings reported here may ultimately only apply to vocational colleges located in comparatively developed regions of China and therefore additional caution should be taken when generalizing them to the entire population of vocational colleges in China. Future studies should either include more regionally equivalent samples or explore how the context may differ across regions as a means to better represent the heterogeneity of China and improve the external validity of the results.

Although information has been gathered on demographic variables, and they have been described for use as a reference about the sample used in this study, they will not be examined within the context of the tested constructs or relationships as they do not relate back to the original constructs. In addition, the research model has been developed based on theory and tested through empirical evidence and not intended to derive causal relationships based upon demographic variables. Future research may look at additional ways to examine demographic variables as predictors or moderators to create additional nuances to the current data.

The cross-sectional nature of this study creates several limitations. The design of this study prevents making causal connections between the variables. While the theoretical relationships established by the COR model and the empirical relationships established by previous studies support the proposed relationships, only using data collected at one point in time does not provide a mechanism to verify the variables occurred prior to each other. Despite being assumed to be a mediator in the relationship between workplace stressors and quiet quitting, the possibility exists that these relationships are reciprocal and as such, disengagement behaviors may also lead to burnout or strengthen burnout over time. Additionally, while PLS-SEM allows estimation of indirect effects, cross-sectional mediation analyses cannot be regarded as establishing causal mediation because they do not provide any temporal separation. Thus, the mediation effects observed in the study reflect theoretically-supported relationships rather than confirmed causal mechanisms. Future researchers are encouraged to use longitudinal or multiple-wave designs to test the temporal dynamics hypothesized in this research. For example, P–O fit at T1; WFC and work overload at T1; burnout measured at T2; and quiet quitting at T3 would all provide longitudinal data. Alternatively, researchers could examine potential reciprocal relationships between quitters and burnout through cross-lagged panel models which would provide stronger evidence for causal ordering and resource loss models over time.

## Data Availability

The original contributions presented in the study are included in the article/supplementary material, further inquiries can be directed to the corresponding author.

## Appendix A

Table A1

**Table A1:** Measurement questionnaire.

Person-organization fit
The things that I value in life are very similar to the things that my organization values.
My personal values match my organization’s values and culture.
My organization’s values and culture provide a good fit with the things that I value in life.
There is a good fit between what my job offers me and what I am looking for in a job.
The attributes that I look for in a job are fulfilled very well by my present job.
The job that I currently hold gives me just about everything that I want from a job.
The match is very good between the demands of my job and my personal skills.
My abilities and training are a good fit with the requirements of my job.
My personal abilities and education provide a good match with the demands that my job places on me.
In general, I feel that I am highly fit with my organization.
Work–family conflict
The demands of my work interfere with my family life.
The amount of time my job takes up makes it difficult to fulfill my family responsibilities.
Things I want to do at home do not get done because of my job demands puts on me.
My job produces strain that makes it difficult to fulfill my family duties.
Due to work-related duties, I have to make changes to my plans for my family activities.
In general, I am experiencing significant work–family conflict.
Work overload
I have been given too many responsibilities in my work.
The amount of work I have to do interferes with the quality of life I want to maintain.
I feel overburdened by my work.
I feel there is a need to reduce some aspects of my work.
In general, my workload is severely overloaded.
Job burnout
I feel emotionally drained from my work
I feel used up at the end of the workday.
I feel tired when I get up in the morning and have to face another day at work.
Working all day is really a strain for me.
I feel burned out from my work.
I have become more callous toward work since I took this job.
I have become less enthusiastic about my work.
I doubt the significance of my work.
I have become more and more indifferent in the contribution of my work.
In general, I feel increasingly burnt out in my work.
Quiet quitting
I am doing the bare minimum work to avoid being fired.
I feel I have a lack of interest in attending meetings.
I often avoid working more hours, if there is no additional pay.
I feel there is a lack of passion and enthusiasm in me to work above and beyond.
I feel there is a lack of opportunities to learn and grow in my college.
I feel there is a lack of meaningfulness at work.
In general, I prefer quiet quitting at my work.
